# Performances of the LBP Based Algorithm over CNN Models for Detecting Crops and Weeds with Similar Morphologies

**DOI:** 10.3390/s20082193

**Published:** 2020-04-14

**Authors:** Vi Nguyen Thanh Le, Selam Ahderom, Kamal Alameh

**Affiliations:** Electronic Science Research Institute, Edith Cowan University, Perth 6000, Australia; s.ahderom@ecu.edu.au (S.A.); k.alameh@ecu.edu.au (K.A.)

**Keywords:** local binary pattern (LBP), k-FLBPCM, deep convolutional neural networks, precision agriculture, crop/weed classification and detection

## Abstract

Weed invasions pose a threat to agricultural productivity. Weed recognition and detection play an important role in controlling weeds. The challenging problem of weed detection is how to discriminate between crops and weeds with a similar morphology under natural field conditions such as occlusion, varying lighting conditions, and different growth stages. In this paper, we evaluate a novel algorithm, filtered Local Binary Patterns with contour masks and coefficient k (k-FLBPCM), for discriminating between morphologically similar crops and weeds, which shows significant advantages, in both model size and accuracy, over state-of-the-art deep convolutional neural network (CNN) models such as VGG-16, VGG-19, ResNet-50 and InceptionV3. The experimental results on the “bccr-segset” dataset in the laboratory testbed setting show that the accuracy of CNN models with fine-tuned hyper-parameters is slightly higher than the k-FLBPCM method, while the accuracy of the k-FLBPCM algorithm is higher than the CNN models (except for VGG-16) for the more realistic “fieldtrip_can_weeds” dataset collected from real-world agricultural fields. However, the CNN models require a large amount of labelled samples for the training process. We conducted another experiment based on training with crop images at mature stages and testing at early stages. The k-FLBPCM method outperformed the state-of-the-art CNN models in recognizing small leaf shapes at early growth stages, with error rates an order of magnitude lower than CNN models for canola–radish (crop–weed) discrimination using a subset extracted from the “bccr-segset” dataset, and for the “mixed-plants” dataset. Moreover, the real-time weed–plant discrimination time attained with the k-FLBPCM algorithm is approximately 0.223 ms per image for the laboratory dataset and 0.346 ms per image for the field dataset, and this is an order of magnitude faster than that of CNN models.

## 1. Introduction

Precision agriculture plays an indispensable role in increasing the productivity of agriculture, food security and sustainability, and reducing the detrimental impacts on the environment. Amongst the major threats to agricultural production are weed infestation, plant diseases and herbicide resistance. Identifying weeds at early crop growth stages brings many benefits for weed management prior to crop damage. This results in a reduction in herbicide usage, minimizes the negative impacts on the environment, improves grower profitability and maintains high crop quality [1]. Variable herbicide application systems, based on weed identification algorithms, have shown great promise in experimental results. A study on the effectiveness of the sensor-based precision herbicide application is described in [2]. The average herbicide savings in 13 field trials was 24.6%, using sensors for detecting weeds [2]. In another four-year study, average herbicide savings for controlling grass weeds were 78% in maize and 36% in sugar beet crops [3]. Furthermore, the amount of herbicide used for controlling broad-leaved weeds was saved by up to 11% in maize and 41% in sugar beet crops [3].

With the technological advances in precision agriculture, a substantial number of studies have been developed to discriminate crops from weeds [4,5,6,7]. One of the most popular and effective methods is plant image analysis [4,8]. There have been many techniques used for analyzing images in the stages of pre-processing, segmentation, feature extraction and classification. Each stage plays an indispensable role in weed detection. However, the performance of computer vision algorithms is greatly dependent on the selection of an appropriate set of features [9]. Particularly, the key characteristics of vegetation (crops and weeds), which comprise biological morphology [10,11,12], spectral features [13,14,15], spatial contexts [16,17,18] and visual textures [19,20,21] can be extracted by applying different characterization methods. Each of these characteristics has its own advantages, and depends on the complexity of the generated datasets for plant species. Machine learning techniques, such as Support Vector Machines (SVM), K-means and Artificial Neural Networks (ANNs) can be applied to classify these species [5,22]. 

One of the most competitive and widespread broadleaf weeds in Australia is wild radish (Raphanus raphanistrum) [23]. Wild radish has a devastating impact on canola crops and farmers have been struggling to effectively eradicate it and minimize its threats to canola crop fields [24]. When the leaf shape of crops and weeds have different morphologies, for example broad leaves and narrow leaves, they can be easily distinguished. However, canola and wild radish broadleaf plants have a very similar color and shape. Datasets collected at different growth stages, rotations, and illuminations for canola, corn, wild radish and soil background have been generated to investigate effective plant discrimination based on the combination of local binary pattern operators (LBP) and multiclass support vector machine methods. However, due to their similar leaf shapes, the classification accuracy was considerably reduced [25]. While LBP is one of the most robust and effective methods for plant classification based on morphology [26,27,28,29,30,31], to overcome the classification limitation when plant species have similar shape and color, additional features must be combined with LBP features. In this paper, we demonstrate the performance of a novel plant classification technique, entitled k-FLBPCM, which is based on the use of plant contour features and filtered LBP features with a coefficient k to improve the accuracy rate of broadleaf plants of close colors and shapes [32]. We also compare our method with other methods that have recently been reported.

Despite many efforts to extract leaf features and classify plants using complex computer vision algorithms [33,34,35,36,37], plant recognition is still regarded as a challenging problem [38]. For the machine-vision-based classification of plant leaves in real field conditions, many challenging problems arise, including lighting conditions, overlapping, occlusion, and damaged leaves. Recently, studies on deep learning (DL) have produced extremely promising classification results for various applications, such as image recognition, natural language processing and speech recognition [39,40]. Within the realm of precision agriculture, a variety of agricultural challenges have been solved by using DL [4]. It is also important to note that DL tools represent a subfield of machine learning, enabling artificial neural networks to automatically extract abstract and robust features that are invariant to illumination and distortions from raw data [4]. Particularly, DL extracts the high level features from the hierarchical layers of data representation by composing lower level features [39]. With a high computing capacity and data, DL techniques combining feature extraction and classification stages can potentially reduce manual and expensive engineering processing, thus making accurate real-time plant classification viable and cost-effective [4].

Convolutional Neural Networks (CNNs) are particularly based on deep learning models, and have been widely used for the image-based classification of plants. CNNs have exhibited high classification accuracies because of the use of spatial information and correlation filters between layers [41,42,43,44,45,46,47]. CNNs typically comprise several layers, namely, convolutional layers, pooling layers and fully connected layers, in addition to activation functions. The convolutional layers are regarded as feature extractors. The role of the pooling layers is to reduce the dimensionality of images, while the fully connected layers are used for classification [48]. CNN architectures have been finely tuned and developed in recent years to allow the reuse of transfer learning. Amongst the popular and successful CNN architectures are AlexNet [49], Visual Geometry Group (VGG) [50], GoogleNet [51], Inception [52] and ResNet [53]. Based on the evolution of the CNN architectures, it is generally observed that the more accurate CNNs tend to have deeper learning. In this paper, we choose VGG-16, VGG-19, ResNet-50 and Inception-V3 architectures that have demonstrated strong performances on various datasets and state-of-the-art results in the ImageNet Large Scale Visual Recognition Challenge (ILSVRC) [50,54], and compare their performances with the k-FLBPCM method.

There have been several comparative studies of CNNs and LBP for image classification [55,56,57], with datasets captured by various devices in different conditions. While the CNNs and LBP performances have been extensively investigated for proof-of-concept classification demonstration, the computation time for both deep learning and machine learning methods was little mentioned. Despite the attractive classification capabilities of CNNs, some limitations still exist, such as the need for huge datasets for the training process, overfitting problems and time execution [4]. In this paper, four well-known DL architectures comprising VGG-16, VGG-19, ResNet-50 and Inception-V3 [52] are used to extract relevant features for the identification of crops and weeds with similar morphological characteristics. The performances of the DL architectures and the proposed machine learning method (k-FLBPCM + SVM) are compared for the detection of crops and weeds of similar morphologies using in the “bccr-segset” dataset, collected in a laboratory setting (published online) and in the “fieldtrip_can_weeds” field dataset (published online now for this paper). The laboratory dataset, which contains 30,000 plant images, was captured at four different growth stages and has four classes including soil background, canola, corn and wild radish. The field dataset, on the other hand, comprises 4914 field images and was gathered under complex field environments and illumination variations (morning and afternoon light). Further, we measure the time typically spent in training and testing of deep neural networks and compare it with that for the k-FLBPCM feature extractor with an SVM classifier.

## 2. Materials and Methods

### 2.1. k-FLBPCM

The LBP method, which was introduced by Ojala et al. in 1996 [58], has long been the most effective and robust texture descriptor in many areas [59,60,61]. The use of the LBP algorithm has many advantages, such as computational, rotation and illumination invariance. LBP was developed to extract dominant features with the aim of enhancing the effectiveness of classification accuracy, and may be combined with other feature extraction methods to improve classification accuracy in various applications [61,62,63].

Specifically, for weed and crop classification using machine vision, the recognition of leaves is based on morphological features, such as texture and shape. Due to the similarity in color for canola and wild radish species, color features cannot be considered in the context of identifying green plants. According to the “bccr-segset” and “fieldtrip_can_weeds” dataset, broadleaf canola and wild radish plants pose challenges for identifying their similar morphology at every growth stage. Therefore, we developed a novel LBP-based algorithm to solve this problem. To be more specific, texture features were extracted by the combination of LBP operators and morphological features were extracted by applying contour masks on plant images. This method is based on combining contour mask features and filtered LBP features with a coefficient k, and is called k-FLBPCM [32]. Due to the independence of morphological features with rotation, different growth stages and geometric translation, the combination of these features enhances the crop/weed classification and discrimination accuracy.

**Figure 1:**
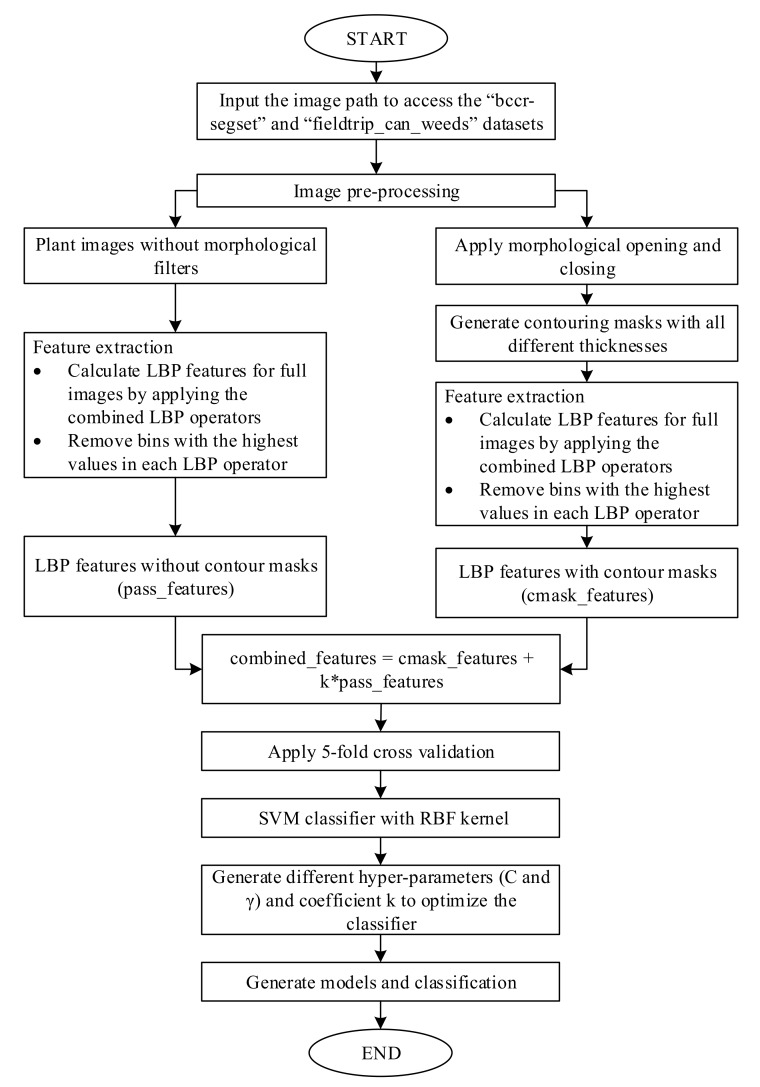
The flowchart presents how the k-FLBPCM algorithm works [32].

The detailed flowchart of the new LBP method is presented in Figure 1. All plant images in datasets were divided into two branches. For the left branch, all images went through the feature extraction stage without applying morphological operators. At the feature extraction process, different LBP operators were combined. In each bin of the generated LBP histogram, the dominant bin value was removed in order to allow a better distribution of features. Hence, the bins with the highest value were removed. From the left branch, LBP features (pass_features) were extracted. For the right branch, opening and closing morphological operators were applied to all images using a 5 × 5 structure element. Before these images were processed by the feature extraction stage, contouring masks were generated from morphological image-processing filters with different thicknesses. Then, these masks were processed by using the combination of LBP operators and removing the bins with the highest values, as done in the left branch. From the right branch, LBP features with contour masks (cmask_features) were extracted. The combined features were calculated by multiplying pass_features with a coefficient k and summing with cmask_features. At the classification stage, a 5-fold cross validation method was applied to prevent overfitting. Then, the SVM classifier with an RBF kernel was used. To achieve higher classification accuracies, hyper-parameters (C and Gamma) were appropriately tuned to attain the maximum accuracy. The source code of this method is presented at the link (https://github.com/vinguyenle/k-FLBPCM-method).

### 2.2. Methods

#### 2.2.1. Data Collection in the Laboratory

**Figure 2:**
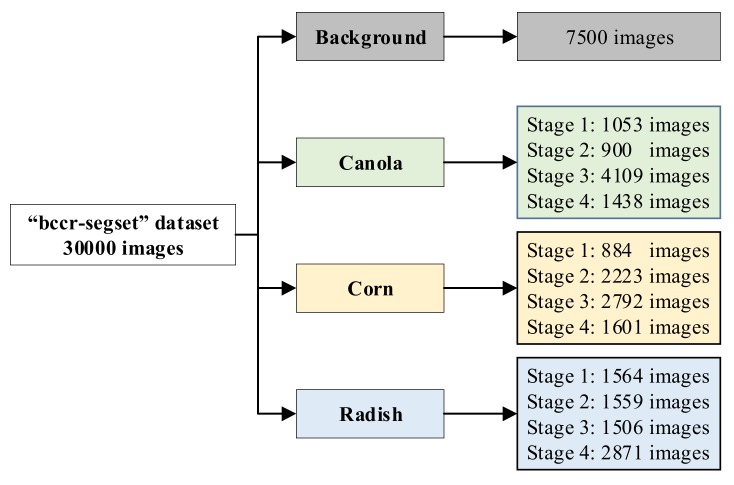
The “bccr-segset” dataset and its four-growth stages.

Plant images were adopted from the “bccr-segset” dataset (published online) [25]. All data were captured on a custom-built testing facility at ESRI (Electron Science Research Institute), Edith Cowan University, Australia [32]. The size of all images was 228 × 228 pixels. As can be seen in Figure 2, the dataset comprises 30,000 plant images partitioned into four classes (canola, corn, wild radish and background) under different rotations, scales and illumination conditions. Images were collected by applying different rotation angles (45°, 90°, 135°, 180°, 225°, 270°, 315°, 360°), lighting conditions (sunlight and fluorescent light), sizes and morphologies of plants through four growth stages [25].

The 5-fold cross validation was used in the “bccr-segset” dataset. This dataset was randomly shuffled and divided into five equal subsets with 6000 plant images in each subset. Then, a model was trained five times; each time, a different subset was used as a testing set with 6000 images, while the remaining four subsets used 24,000 images. In other words, each testing set was generated in each iteration, until each fold in five folds has been used as the testing set. In addition, as for deep neural networks, 24,000 images were divided into two datasets including 21,000 images for training and 3000 images for validation.

**Figure 3:**
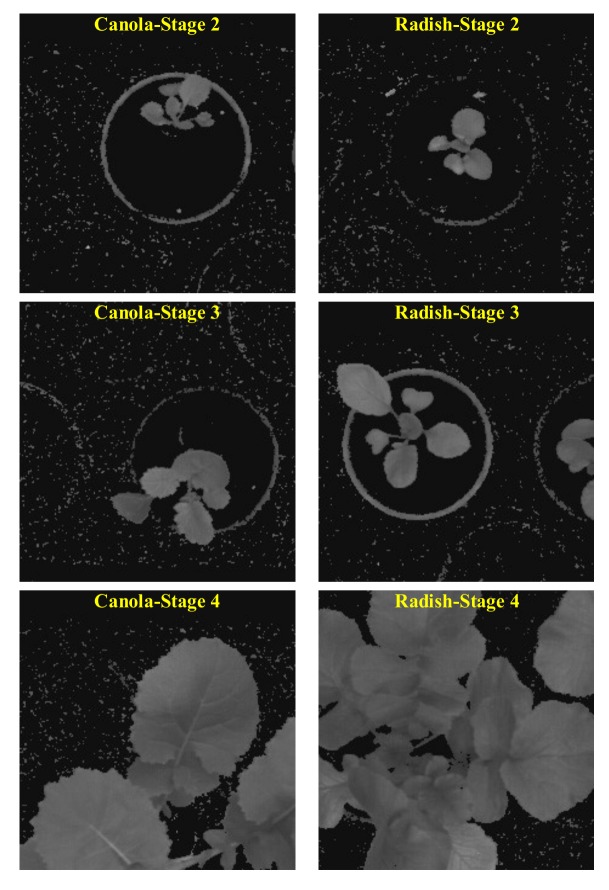
Canola and radish plants at different growth stages in the “bccr-segset” dataset.

Based on the “bccr-segset” dataset collected at different growth stages, we tested the performance of the k-FLBPCM method and CNNs when training and testing sets had the same growth stage and different growth stages. As can be seen in Figure 3, the size of both canola and radish plants at the fourth stage is larger than in the image frame, while the full size of canola and radish plants at the second and third stage can be observed. We used the training set at the fourth stage and the testing set at the second stage. However, since the performance of both the k-FLBPCM method and CNNs was unsatisfactory, we selected the canola and radish images at the second and third growth stages extracted from the “bccr-segset” dataset (online) as follows:

- 1600 images (800 canola images and 800 radish images) at stage 3 for training;
- 400 images (200 canola image and 200 radish images) at stage 3 for testing;
- 400 images (200 canola image and 200 radish images) at stage 2 for testing.

**Figure 4:**
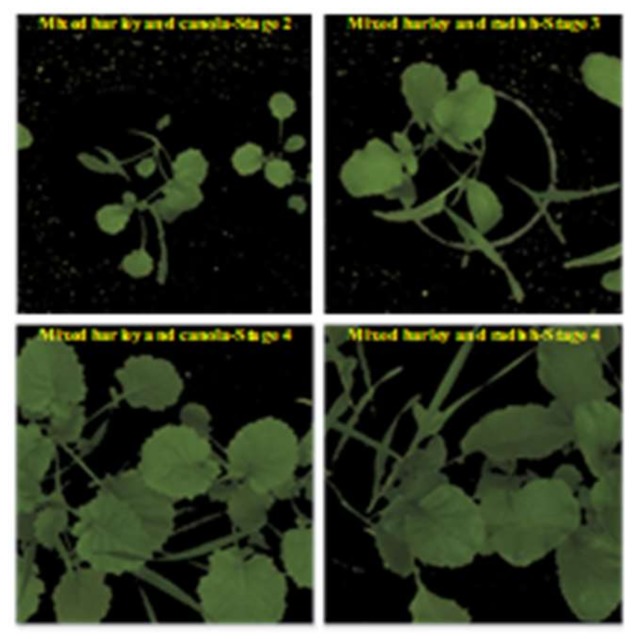
Barley was mixed with canola and wild radish at different growth stages.

We also collected another dataset, called “mixed-plants” dataset (online), to validate the performance of the k-FLBPCM method and CNN models. For this dataset, barley, canola and wild radish were mixed together and then grown in plant pots. There were two groups including a 50:50 barley:canola mixture, and a 50:50 barley:wild radish mixture. These groups were captured at different growth stages, as can be seen in Figure 4**.** The corresponding dataset comprised:

- 3000 images (1500 mixed barley-canola images and 1500 mixed barley-radish images) at stage 4 for training;
- 750 images (375 mixed barley-canola images and 375 mixed barley-radish images) at stage 4 for testing;
- 750 images at both stage 2 and stage 3 (375 mixed barley-canola images at stage 2 and 375 mixed barley-radish images at stage 3) for testing.

#### 2.2.2. Field Data Collection

**Figure 5:**
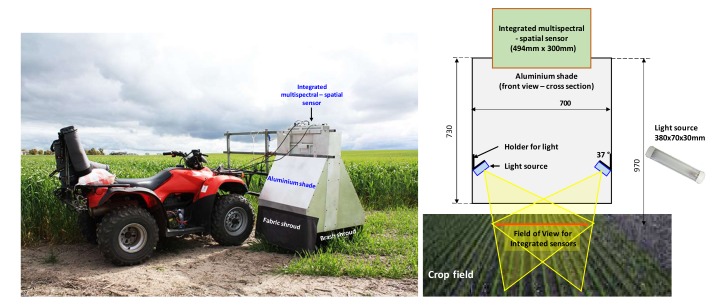
An integrated weed sensing system to collect plant images in the field.

Field images were captured by an integrated weed sensing system with the combination of multispectral and spatial sensors at a commercial farm in Cunderdin, Western Australia, shown in Figure 5**.** This hardware system, which is housed at the Electron Science Research Institute (ESRI), Edith Cowan University, Australia, consists of two components (i) a Xilinx Zynq ZC702 development board with a VITA 2000 camera sensor and (ii) a Plan Discrimination Unit (PDU) [15] based on spectral reflectance measurements.

**Figure 6:**
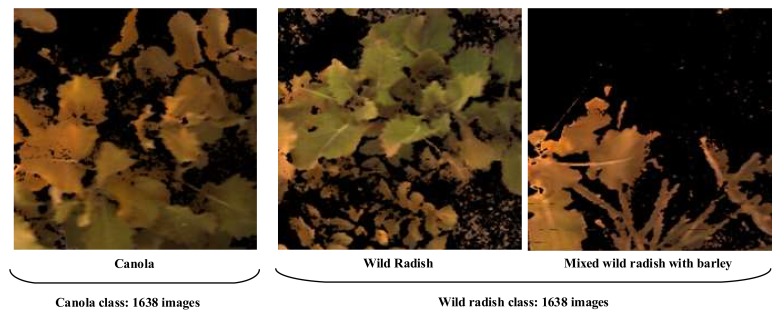
Segmented canola and wild radish classes under complex field environments.

We collected a “fieldtrip_can_weeds” dataset (published online) under different weather conditions (cloudy, windy, and sunny) and illumination variations (morning and afternoon light). There are 4914 field images with three classes, including background (1638 images), canola (1638 images), wild radish (1638 images). When all field images were segmented by using Excess Green minus Excess Red Indices (ExG-ExR) method, the segmented plants were presented in Figure 6. It is worth noting that mixing wild radish and barley in the wild radish class under practical field conditions is to challenge our algorithm and DCNN models. 

With the aim of comparing the various weed detection methods, we set up experiments with similar conditions. Therefore, the dataset was divided into five roughly equal parts. In particular, with the 5-fold cross validation method, a model was trained five times; each time, a different single part was used as a testing set, with 982 field images, while the remaining four parts with 3928 field images were used for training. Then, the cross-validation process was repeated five times, with each testing set used only once. As for deep neural networks, it is important to note that 3928 field images were divided into two datasets, including 3437 images for training and 491 images for validation.

#### 2.2.3. Training k-FLBPCM and CNNs Models

Before training, to meet the input dimension requirement of deep neural networks, all plant images were resized. In this paper, the input shape of the VGG-16, VGG-19 or Resnet-50 networks was 224 × 224 × 3 pixels, while the input shape of the Inception-V3 network was 299 × 299 × 3 pixels. It is important to note that all generated models, training and testing processes were implemented using the deep learning framework, Keras (with TensorFlow 2.0 backend). The Ubuntu 18.04 LTS operating system and Python 3.7 were used in this paper. The training and testing were performed on a workstation with an Intel Core i7-7820X CPU, a GeForce GTX1080Ti Graphic Processing Unit (GPU) with 11GB of memory. Additionally, the k-FLBPCM method was also implemented on the same machine to compare with CNN models.

When deep neural networks were trained on natural images, the features learned in the first layers tended to be general and then had transitions to be more specific by the last layers of the network. Thus, transfer learning was regarded as an efficient technique to transfer features learned in one or more datasets and reuse these features to improve learning in other datasets [64]. In other words, the transfer learning method was responsible for keeping the parameters of the previous layers, then removing the last layer of CNN models, and then retraining the last layer. In this paper, we chose the VGG-16, VGG-19, ResNet-50 and Inception V3 models, which were fine-tuned by using neural networks pre-trained on the ILSVRC versions of the ImageNet dataset. Such CNNs are suitable for transfer learning in network-based, deep transfer learning [64]. The public ImageNet dataset consists of 1.28 million natural images and 1000 classes corresponding to 1000 categories. Therefore, the last layer in this dataset has 1000 output nodes. To apply for the “bccr-segset” dataset collected from the laboratory, we changed the output to four output nodes corresponding to four categories (background, canola, corn and wild radish). For the “fieldtrip_can_weeds” dataset collected from the field, the output was changed to three output nodes corresponding to three categories (background, canola, and wild radish). 

As for the aforementioned CNN models, each model was loaded with the corresponding weights pre-trained on the ImageNet dataset and resized plant images to the standard image size, before the training process, as shown in Table 1**.** Then, we used stochastic gradient descent (SGD) to optimize parameters over a training set using mini-batches of 32 and 64 images, and selected dropout rates of 50% and 20% in the training stage for regularization. After some preliminary training experiments, the learning rate was adjusted to 0.001 and the number of epochs was set to 10 for the laboratory and field datasets. VGG-16 and VGG-19 models were kept as the original models, and changed to four outputs in the last layer for the laboratory dataset and three outputs for the field dataset, while ResNet-50 and Inception-V3 models were fine-tuned by adding a max pooling layer with a pool size 5 × 5, a flatten layer, a fully connected layer with a dense 1024 and ReLU activation and the last layer with outputs (four and three outputs for the laboratory and field dataset, respectively) and softmax activation. It is observed that these added layers show good performances with our dataset.

## 3. Results and Discussion

We conducted three comparison experiments to investigate the performances of the k-FLBPCM method and CNNs on the laboratory and field datasets.

### 3.1. Comparison of the Classification Accuracies of the k-LBPCM Method and Deep Neural Networks in the “Bccr-Segset” Dataset

The “bccr-segset” dataset (comprising 30,000 plant images) was equally divided into four classes (background, canola, corn and wild radish). We applied five-fold cross validation on the dataset to prevent overfitting. The dataset was randomly shuffled and one of the five folds was taken as the test set, while the remaining folds were considered as the training set. The random splits for each fold were performed using random seeds.

Before training, each model was loaded with the corresponding weights that were pre-trained on ImageNet. Then, we used the transfer learning technique to fine tune models, as described in Section 2 (Materials and Method). The standard *sparse_categorial_crossentropy* loss function was used for training. After the trial with using optimizers, the SGD optimizer was selected due to its superior performance. The momentum was 0.9 and the learning rate was 0.001. The optimal batch size and dropout of the training set were selected experimentally. The observed average classification accuracies varied across the different models.

The accuracy obtained for the k-FLBPCM method was 98.60% with C = 30, γ = 10^−5^, thickness = 2 and coefficient k = 0.2. The classification accuracies of deep neural networks were slightly higher than the k-FLBPCM method, as shown in Table 2. Furthermore, VGG-16, VGG-19 and Inception-V3 models attained higher average accuracies than that of the ResNet-50 model. As can be seen in Table 3, confusion matrices of the test set for individual classes in the “bccr-segset” dataset were presented to compare the performance of the selected methods in distinguishing cultivated plants from weeds with a similar appearance.

With the batch size of 32 and dropout 0.2, the small discrepancies between the classification accuracies of the k-FLBPCM method and CNN models were observed in Table 4. In addition, the performance of the VGG-16 model was the highest, achieving 99.87% in the “bccr-segset” dataset.

In order to explore the influence of batch size on the stability of the learning process, the next experiment kept all parameters and changed the batch size from 32 images to 64 images. The average classification accuracies of the VGG-16 and Inception-V3 models were higher than other selected neural networks and the k-FLBPCM method, as shown in Table 5. It is clear from Table 2, Table 4 and Table 5 that the changes in the average classification accuracy of the methods were insignificant when the batch size was increased from 32 to 64.

### 3.2. Comparison of the Classification Accuracies of the k-LBPCM Method and Deep Neural Networks in the Training and Test Sets of Different Growth Stages

In the previous experiments, plant images with different growth stages were shuffled randomly in five folds. This means that the features were learned through the training process. As for the k-FLBPCM method, it learned the features of leaf shapes, especially the morphology of canola and radish plants. For the deep neural networks, we suspected that the learned features might not be extracted from the edges of the canola and radish broadleaves. Therefore, an experiment was conducted to compare the performance of these methods with the training and testing sets from different growth stages. 

Due to the superior performance of VGG-16 and Inception-V3 in the previous experiments, these models were selected for comparison with the k-FLBPCM method in this experiment. The transfer learning technique was applied again to reduce the training time and effort required to recognize weeds and crops, and efficiently reuse the generated general features. The layers of VGG-16 model remain unchanged and the last layer was changed from 1000 outputs (ImageNet) to two outputs (canola and radish plants in the “bccr-segset” dataset). Next, the Inception-V3 model was fine-tuned by adding some custom layers, including, a max pooling layer with a pool size 5 × 5, a flatten layer, a fully connected layer with a dense 1024 nodes and ReLU activation and a last layer with two outputs and softmax activation. Dropout was set to 0.5 for both VGG-16 and Inception-V3 models.

As discussed in Section 2, the first experiment used 1600 images (800 canola images and 800 radish images) collected at stage 3 for training and 400 images (200 canola image and 200 radish images) collected at stage 3 for testing. In the second experiment we reused the 1600 images (800 canola images and 800 radish images) collected at stage 3 for training and used 400 images (200 canola image and 200 radish images) collected at stage 2 for testing. We used the ratio 80:20 for the sizes of the training and test sets. The remaining 20% of plant images were reserved for testing and not used in the training process. A SGD optimizer with a learning rate of 0.001, a learning decay of 0.001 and a momentum of 0.9, was used in the “bccr-segset” dataset of canola and radish plants. The impact of the different batch sizes (32 and 64 images) on the accuracy of the networks during training in the “bccr-segset” dataset was not substantial, as discussed in Section 3.1. Hence, a batch size of 32 images was adequate, and used in this experiment with 30 epochs and a dropout of 0.5. 

The experimental results shown in Table 6 illustrate the recognition performances of the k-FLBPCM method, VGG-16 and Inception-V3 using training and testing sets, for similar and different growth stages. As for the training and testing sets for similar growth stages (stage 3), the accuracies of VGG-16, Inception V3 and k-FLBPCM methods were relatively similar. It is worth noting that the parameters of the k-FLBPCM method, including C = 100, γ =10^−7^, thickness = 2, and k = 0.2, achieved an accuracy of 97.25%. Using stage 3 in the training set and stage 2 in the testing set, the accuracy of the k-FLBPCM method was 96.75% with parameters C = 100, γ =10^−6^, thickness = 2, and k = 0.2, while the optimal accuracies of VGG-16 and Inception-V3 models dropped to 62.5% (at epoch 18) and 63.8% (at epoch 16), respectively. When the training and testing sets were assigned to different growth stages, the capability of the k-FLBPCM method in recognizing canola and radish plants was significantly higher than those of the VGG-16 and Inception-V3 models. It can be explained that the k-FLBPCM method concentrates on extracting unique features of leaf shapes to train with, whereas VGG-16 and Inception-V3 architectures focus on filtering a wide range of features in plant images through many convolutional layers. Thus, the k-FLBPCM method can identify canola and wild radish plants much more generally than the widespread CNN methods.

To confirm the ability of the k-FLBPCM method to recognize canola and radish plants with high accuracy using the “mixed-plants” dataset, close to that attained using the “bccr-segset” dataset. We conducted another experiment, where barley plants were mixed with canola and radish plants, thus making plant discrimination more challenging. The training and testing data division of the mixed-plants dataset are described in Section 2. A total of 3000 mixed-plant images, collected at the fourth stage, were used for training. Then, 750 mixed-plant images, collected at the fourth stage, were assigned for the test set and another 750 mixed-plant images, collected at the second and third stages, were used for another test set. It is important to note that all images in the test sets were not used for training. However, the training set was combined with each test set, in order to compare the plant discrimination performance of the selected methods.

Table 7 shows the plant classification accuracies for the selected methods. The performance of the VGG-16, Inception-V3 and k-FLBPCM methods for the training and test sets collected at the fourth stage had approximately similar accuracies, namely 100% (at epoch 15), 99.05% (at epoch 30) and 99.73%, respectively. The optimum parameters of the k-FLBPCM method were C = 30, γ =10^−7^, thickness = 2, and coefficient k = 0.2. However, as observed from Table 7**.** for the training set using mixed-plant images collected at the fourth stage and the testing set using images collected at the second and third stages, the k-FLBPCM method again outperformed the CNN methods. The accuracies of VGG-16 and Inception-V3 models were 94.70% (at epoch 13) and 87.36% (at epoch 30), respectively, which are considerably lower than the 99.33% accuracy attained by the k-FLBPCM method.

After these two experiments, it was concluded that the k-FLBPCM method maintains a high accuracy in recognizing single plants or mixed plants when the size of plant images in the training set are bigger than the ones in the test set, even when plant images are collected at different growth stages, whereas, for the same conditions, the accuracies attained by deep neural networks drop to impractical levels. The effectiveness of the k-FLBPCM method is in its ability to identify plant species using images collected at earlier growth stages, even if the available data are insufficient for training.

The k-FLBPCM algorithm provides better recognition accuracy with both the canola–radish subset, from the “bccr-segset” dataset, and the “mixed-plants” dataset. While the selected CNN models were applied to learn features of plants at the fourth growth stage and then identify plants at smaller growth stages (the second and third stages), their classification accuracy was lower than that of the k-FLBPCM algorithm. This is because the combination of extractors, including LBP features and contouring mask features, in the k-FLBPCM algorithm, was able to accurately extract the edges of canola and radish leaves, and this is the key advantage of the k-FLBPCM method, especially with datasets comprising insufficient plant images.

### 3.3. Comparison of the Classification Accuracies of the k-LBPCM Method and DCNNs in the Dataset under Complex Field Conditions

The experiments on the “fieldtrip_can_weeds” dataset were similar to those conducted on the “bccr-segset” dataset. The learning rate was 0.001, the dropout was 0.5, and the output of the CNN models was three output nodes, corresponding to three classes (background, canola and wild radish). As can be seen from Table 8, the classification accuracy obtained for the k-FLBPCM method was 90.94% with C = 1000, γ = 10^−8^, thickness = 2 and coefficient k = 0.5. The accuracies of CNN models (except for VGG−16) were slightly lower than the k-FLBPCM method, indicating the efficacy of the novel algorithm. The experimental results demonstrate the ability of our algorithm to detect canola (crop) and mixed wild-radish–barley (weed) with similar morphology under practical field conditions, compared to the CNN models. However, we expect that the CNNs may achieve higher accuracies when big data are input in the networks. 

### 3.4. Comparison of Execution Times

In precision agriculture, the processing time is an important aspect for real-time operation at practical farming speeds. In addition to the measured accuracies of the VGG-16, Inception-V3 and k-FLBPCM methods reported in the sections mentioned above, both the model training and testing times were measured. 

#### 3.4.1. Training Time

The VGG-16, VGG-19, ResNet-50, Inception-V3 and k-FLBPCM models were implemented on the GPU GTX1080Ti in order to compare their processing times. Table 9 shows the measured total training time periods for all models. The number of epochs was set to 10 and the batch size was 32 images. The training time consumed in each epoch was accumulated from the five folds for all models in the “bccr-segset” and “fieldtrip_can_weeds” datasets. With both datasets, the total training time of the k-FLBPCM model was shorter than that of the VGG-16, VGG-19, ResNet-50 and Inception-V3 models. Note that the time taken to perform the required pre-processing steps was also included in the total training time periods shown in Table 9**.** Particularly, these steps consist of loading plant images, properly resizing them for input to deep neural networks, and applying morphological operators for the k-FLBPCM method.

#### 3.4.2. Testing Time

Table 10 shows the average testing time, which was computed by averaging the testing time periods for the five test folds, and the testing time per image, calculated by dividing the average testing time by the number of images in the “bccr-segset” test set (6000 plant images) and the “fieldtrip_can_weeds” test set (982 field images). As shown in Table 10, the testing time of the k-FLBPCM method was 0.223 ms per image in the “bccr-segset” laboratory dataset, which is more than one order of magnitude shorter than the testing times for the VGG-16, VGG-19, ResNet-50 and InceptionV3, which were 2.667 ms, 3.033 ms, 2.333 ms, and 3.5 ms, respectively. Similarly, the high efficiency of the k-FLBPCM algorithm was also demonstrated in the “fieldtrip_can_weeds” field dataset, where only 0.346 ms per image was necessary to run the field test set by applying our algorithm, compared to the testing time of CNN models. 

Note that the Inception-V3 model requires a longer time in comparison with the other CNN networks, since its architecture is deeper. On the other hand, the k-FLBPCM algorithm has the ability to rapidly extract dominant features due to its computational efficiency. Although the selected deep neural networks eliminate the manual search for good feature extractors through the automatic learning of relevant features, deep neural networks go through many convolutional layers and are susceptible to a high computational complexity. 

It is important to note that the deep-learning-based approaches typically require a large amount of data to outperform the k-FLBPCM method. This explains why the performance of selected neural networks was slightly better than the k-FLBPCM method in recognizing morphologically similar crops and weeds. When pre-trained CNN models are used to train plant images at four different growth stages in the “bccr-segset” dataset, they learn relevant features at each stage. The ability of CNN models is demonstrated by having high sample counts in the dataset and corresponding ground truth annotations. However, for real-time operation at high vehicular speeds, the long image processing time of these complex models makes them impractical if a high-accuracy performance cannot be compromised.

## 4. Conclusions

In this work, we have compared the performances of selected Convolutional Neural Network (CNN) models (VGG-16, VGG-19, ResNet-50 and Inception-V3 models) with the k-FLBPCM algorithm, specifically in identifying crop and weed species of similar morphologies. Experimental results, using the “bccr-segset” laboratory dataset, have shown that the both the CNN models with fine-tuned hyper-parameters and the k-FLBPCM method can achieve classification accuracies close to 99%. With the “fieldtrip_can_weeds” field dataset under complex field environments, the k-FLBPCM method can attain up to 90.94% classification accuracy, compared to the 89.55%, 89.73% and 90.87% accuracies of VGG-19, ResNet-50 and Inception-V3, respectively (except for VGG-16 with 91.55%). However, for effective feature learning, these CNN models require a huge number of plant images to be collected at each of the various growth stages. On the other hand, we have demonstrated that the k-FLBPCM method can identify smaller leaf shapes using images collected at the second and third growth stages, with training using images of large leaves collected at the fourth growth stage. Results have shown that the k-FLBPCM method can achieve a canola–radish discrimination accuracy of 96.75% using the subset generated from the “bccr-segset” dataset, while the accuracies of the VGG-16 and Inception-V3 are 62.50% and 63.80%, respectively. Additional experimental results, using the “mixed-plants” dataset, have demonstrated the effectiveness of the k-FLBPCM method with 99.33% accuracy, whereas the accuracies of the VGG-16 and InceptionV3 are 94.70% and 87.36%, respectively. Furthermore, experimental results have shown that the k-FLBPCM model implemented on the GPU GTX1080Ti requires approximately 0.223 ms per image in the “bccr-segset” laboratory dataset and 0.346 ms per image in the “fieldtrip_can_weeds” field dataset for weed identification and detection. These results show the effectiveness of this algorithm for real-time precision agricultural applications 

It is important to note that choosing an appropriate weed detection method depends on whether real-time operation is required and the detection accuracy can be compromised. The combination of extractors in the k-FLBPCM method can especially work well when the edges of crop and weed leaves can be extracted accurately. On the other hand, CNN models may be a better choice for applications requiring automatic feature extraction, which can be achieved through the convolutional operators.

## Figures and Tables

**Table 1:** Input image sizes used for the CNN and k-FLBPCM models, for the laboratory and field datasets.

Methods	Image Size
k-FLBPCM	228 × 228
VGG-16	224 × 224
VGG-19	224 × 224
ResNet-50	224 × 224
Inception-V3	299 × 299

**Table 2:** Classification accuracies of the test set, in the “bccr-segset” dataset, for different methods, for a batch size of 32 and dropout 0.5.

Methods	Accuracy of the Testing Set					
	Fold 1	Fold 2	Fold 3	Fold 4	Fold 5	Average Accuracy
k-FLBPCM	98.67%	98.75%	98.60%	98.56%	98.41%	98.60%
VGG-16	99.83%	99.73%	99.75%	99.90%	99.85%	99.81%
VGG-19	99.82%	99.22%	99.82%	99.52%	99.85%	99.65%
ResNet-50	99.48%	99.58%	99.62%	99.72%	99.67%	99.61%
Inception-V3	99.83%	99.75%	99.55%	99.85%	99.92%	99.78%

**Table 3:** Confusion matrices of the test set, in the “bccr-segset” dataset, for different methods, for a batch size of 32 and dropout 0.5.

Methods	Classes	Background	Canola	Corn	Radish
**k-FLBPCM**	Background	1497	1	0	0
	Canola	0	1457	4	38
	Corn	0	3	1495	0
	Radish	0	37	0	1461
**VGG-16**	Background	1484	0	0	0
	Canola	0	1491	0	1
	Corn	0	0	1494	0
	Radish	0	5	0	1525
**VGG-19**	Background	1483	0	0	0
	Canola	2	1519	0	5
	Corn	2	0	1495	0
	Radish	0	0	0	1494
**ResNet-50**	Background	1483	1	0	0
	Canola	0	1490	0	2
	Corn	1	2	1491	0
	Radish	0	11	0	1519
**Inception-V3**	Background	1483	0	0	0
	Canola	0	1524	1	1
	Corn	2	0	1495	0
	Radish	0	1	0	1493

**Table 4:** Classification accuracies of the test set, in the “bccr-segset” dataset, for different methods, for a batch size of 32 and dropout 0.2.

Methods	Accuracy of the Testing Set					
	Fold 1	Fold 2	Fold 3	Fold 4	Fold 5	Average Accuracy
k-FLBPCM	98.67%	98.75%	98.60%	98.56%	98.41%	98.60%
VGG-16	99.80%	99.85%	99.87%	99.93%	99.92%	99.87%
VGG-19	99.80%	99.83%	99.85%	99.85%	99.90%	99.85%
ResNet-50	99.82%	99.82%	99.22%	98.92%	99.25%	99.41%
Inception-V3	99.65%	99.72%	99.62%	99.65%	99.60%	99.65%

**Table 5:** Classification accuracies of the test set among different methods in the “bccr-segset” dataset with the batch size of 64 and dropout 0.2.

Methods	Accuracy of the Testing Set					
	Fold 1	Fold 2	Fold 3	Fold 4	Fold 5	Average Accuracy
k-LBPCM	98.67%	98.75%	98.60%	98.56%	98.41%	98.60%
VGG-16	99.82%	99.78%	99.90%	99.63%	99.85%	99.80%
VGG-19	99.73%	99.78%	99.83%	99.53%	98.82%	99.54%
ResNet-50	99.65%	99.52%	99.10%	99.70%	99.70%	99.53%
Inception-V3	99.82%	99.68%	99.83%	99.82%	99.85%	99.80%

**Table 6:** Comparison of the classification accuracies of methods in the use of canola and radish plants at different growth stages in the “bccr-segset” dataset.

Methods	Canola and Radish in the “Bccr-Segset” Dataset	
	Train-Stage3 and Test-Stage3	Train-Stage3 and Test-Stage2
	Test Accuracy	Test Accuracy
k-FLBPCM	97.25%	**96.75%**
VGG-16	98.96%	62.50%
Inception-V3	97.92%	63.80%

**Table 7:** Comparison of the classification accuracies of the VGG-16, Inception-V3 and k-FLBPCM methods when mixed-barley–canola and mixed-barley–radish images collected at different growth stages are used for the dataset.

Methods	“Mixed-Plants” Dataset	
	Train-Stage4 and Test-Stage4	Train-Stage4 and Test-Stage2 and Stage3
	Test Accuracy	Test Accuracy
k-FLBPCM	99.73%	99.33%
VGG-16	100%	94.70%
Inception-V3	99.05%	87.36%

**Table 8:** Classification accuracies of the test set, in the “fieldtrip_can_weeds” dataset, for different methods, for a batch size of 32 and dropout 0.5.

Methods	Accuracy of the Testing Set					
	Fold 1	Fold 2	Fold 3	Fold 4	Fold 5	Average Accuracy
k-FLBPCM	92.33%	91.33%	90.18%	90.54%	90.34%	90.94%
VGG16	91.34%	91.55%	91.55%	91.75%	91.55%	91.55%
VGG19	90.12%	91.04%	89.41%	89.71%	87.47%	89.55%
Resnet50	88.59%	90.53%	90.94%	89.10%	89.51%	89.73%
Inceptionv3	91.75%	90.73%	91.04%	89.10%	91.75%	90.87%

**Table 9:** Total training time of the k-FLBPCM and the VGG-16, VGG-19, ResNet-50 and Inception-V3 models for datasets in the laboratory and in the field.

	Bccr-Segset Dataset	Fieldtrip_Can_Weeds Dataset
Methods	Total Training Time (Second)	Total Training Time (Second)
k-LBPCM	901.2	165.9
VGG-16	8692	1394
VGG-19	10003	1563
ResNet-50	7657	1483
Inception-V3	11014	1907

**Table 10:** Testing time of the k-FLBPCM method and CNNs for the laboratory dataset (6000 images used for the test set) and the field dataset (982 images used for the field test set).

**Bccr-Segset Dataset (In the Laboratory)—Test**		
**Methods**	**Average Testing Time (Second/Test Set)**	**Testing Time (Millisecond/Image)**
k-LBPCM	1.34	0.223
VGG-16	16	2.667
VGG-19	18.2	3.033
ResNet-50	14	2.333
Inception-V3	21	3.500
**Fieldtrip_Can_Weeds Dataset (In the Field)**		
k-LBPCM	0.34	0.346
VGG-16	3	3.055
VGG-19	3.2	3.259
ResNet-50	3	3.055
Inception-V3	4.6	4.684

## References

[1] Aitkenhead M., Dalgetty I., Mullins C., McDonald A.J.S., Strachan N.J.C. (2003). Weed and crop discrimination using image analysis and artificial intelligence methods. Comput. Electron. Agric..

[2] Dammer K.-H., Wartenberg G. (2007). Sensor-based weed detection and application of variable herbicide rates in real time. Crop Prot..

[3] Gerhards R., Christensen S. (2003). Real-time weed detection, decision making and patch spraying in maize, sugarbeet, winter wheat and winter barley. Weed Res..

[4] Kamilaris A., Prenafeta-Boldú F.X. (2018). Deep learning in agriculture: A survey. Comput. Electron. Agric..

[5] Liakos K., Busato P., Moshou D., Pearson S., Bochtis D. (2018). Machine learning in agriculture: A review. Sensors.

[6] Pallottino F., Biocca M., Nardi P., Figorilli S., Menesatti P., Costa C. (2018). Science mapping approach to analyze the research evolution on precision agriculture: World, EU and Italian situation. Precis. Agric..

[7] Pallottino F., Menesatti P., Figorilli S., Antonucci F., Tomasone R., Colantoni A., Costa C. (2018). Machine vision retrofit system for mechanical weed control in precision agriculture applications. Sustainability.

[8] Wang A., Zhang W., Wei X. (2019). A review on weed detection using ground-based machine vision and image processing techniques. Comput. Electron. Agric..

[9] Torralba A., Efros A.A. Unbiased Look at Dataset Bias. Proceedings of the CVPR.

[10] Slaughter D., Giles D.K., Downey D. (2008). Autonomous robotic weed control systems: A review. Comput. Electron. Agric..

[11] Brown R.B., Noble S.D. (2005). Site-specific weed management: Sensing requirements—What do we need to see?. Weed Sci..

[12] Bakhshipour A., Jafari A. (2018). Evaluation of support vector machine and artificial neural networks in weed detection using shape features. Comput. Electron. Agric..

[13] AlSuwaidi A., Veys C., Hussey M., Grieve B., Yin H. Hyperspectral Selection Based Algorithm for Plant Classification. Proceedings of the 2016 IEEE International Conference on Imaging Systems and Techniques (IST).

[14] Herrmann I., Shapira U., Kinast S., Karnieli A., Bonfil D. (2013). Ground-level hyperspectral imagery for detecting weeds in wheat fields. Precis. Agric..

[15] Symonds P., Paap A., Alameh K., Rowe J., Miller C. (2015). A real-time plant discrimination system utilising discrete reflectance spectroscopy. Comput. Electron. Agric..

[16] Midtiby H.S., Åstrand B., Jørgensen O., Jørgensen R.N. (2016). Upper limit for context–based crop classification in robotic weeding applications. Biosyst. Eng..

[17] Wu X., Xu W., Song Y., Cai M. (2011). A detection method of weed in wheat field on machine vision. Procedia Eng..

[18] Liu H., Lee S.H., Saunders C. (2014). Development of a machine vision system for weed detection during both of off-season and in-season in broadacre no-tillage cropping lands. Am. J. Agric. Biol. Sci..

[19] Bakhshipour A., Jafari A., Nassiri S.M., Zare D. (2017). Weed segmentation using texture features extracted from wavelet sub-images. Biosyst. Eng..

[20] Bharati M.H., Liu J.J., MacGregor J.F. (2004). Image texture analysis: Methods and comparisons. Chemom. Intell. Lab. Syst..

[21] Lottes P., Hoeferlin M., Sander S., Müter M., Schulze P., Stachniss L.C. An Effective Classification System for Separating Sugar Beets and Weeds for Precision Farming Applications. Proceedings of the 2016 IEEE International Conference on Robotics and Automation (ICRA).

[22] Nieuwenhuizen A., Tang L., Hofstee J., Müller J., Van Henten E. (2007). Colour based detection of volunteer potatoes as weeds in sugar beet fields using machine vision. Precis. Agric..

[23] Peter N., Mike W., John C. (2014). Wild Radish Management and Strategies to Address Herbicide Resistance.

[24] Lemerla D. (2002). Wild radish threatens canola yields: Mixed methods the answer by Alec Nicol. Ground Cover. https://grdc.com.au/resources-and-publications/groundcover/ground-cover-issue-42-wa/wild-radish-threatens-canola-yields-mixed-methods-the-answer-by-alec-nicol.

[25] Le V.N.T., Apopei B., Alameh K. (2019). Effective plant discrimination based on the combination of local binary pattern operators and multiclass support vector machine methods. Inf. Process. Agric..

[26] Guo Z., Zhang L., Zhang D. (2010). A completed modeling of local binary pattern operator for texture classification. IEEE Trans. Image Process..

[27] Heikkilä M., Pietikäinen M., Schmid C. (2009). Description of interest regions with local binary patterns. Pattern Recognit..

[28] Dubey S.R., Jalal A.S. Detection and Classification of Apple Fruit Diseases Using Complete Local Binary Patterns. Proceedings of the 2012 Third International Conference on Computer and Communication Technology.

[29] Waghmare H., Kokare R., Dandawate Y. (2016). Detection and Classification of Diseases of Grape Plant Using Opposite Colour Local Binary Pattern Feature and Machine Learning for Automated Decision Support System. Proceedings of the 2016 3rd International Conference on Signal Processing and Integrated Networks (SPIN).

[30] Nanni L., Lumini A., Brahnam S. (2012). Survey on LBP based texture descriptors for image classification. Expert Syst. Appl..

[31] Pietikäinen M., Zhao G. (2015). Two decades of local binary patterns: A survey. Advances in Independent Component Analysis and Learning Machines.

[32] Le V.N.T., Ahderom S., Apopei B., Alameh K. (2020). A novel method for detecting morphologically similar crops and weeds based on the combination of contour masks and filtered local binary pattern operators. Giga Sci..

[33] Charters J., Wang Z., Chi Z., Tsoi A.C., Feng D.D. Eagle: A Novel Descriptor for Identifying Plant Species Using Leaf Lamina Vascular Features. Proceedings of the 2014 IEEE International Conference on Multimedia and Expo Workshops (ICMEW).

[34] Cope J.S., Remagnino P., Barman S., Wilkin P. The Extraction of Venation from Leaf Images by Evolved Vein Classifiers and Ant Colony Algorithms. Proceedings of the International Conference on Advanced Concepts for Intelligent Vision Systems.

[35] Kadir A., Nugroho L.E., Susanto A., Santosa P.I. (2013). Leaf classification using shape, color, and texture features. arXiv.

[36] Kumar N., Belhumeur P.N., Biswas A., Jacobs D.W., Kress W.J., Lopez I.C., Soares J.V. (2012). Leafsnap: A computer vision system for automatic plant species identification. European Conference on Computer Vision.

[37] Beghin T., Cope J.S., Remagnino P., Barman S. Shape and Texture Based Plant Leaf Classification. Proceedings of the International Conference on Advanced Concepts for Intelligent Vision Systems.

[38] Lee S.H., Chan C.S., Wilkin P., Remagnino P. Deep-Plant: Plant Identification with Convolutional Neural Networks. Proceedings of the 2015 IEEE International Conference on Image Processing (ICIP).

[39] LeCun Y., Bengio Y., Hinton G. (2015). Deep learning. Nature.

[40] LeCun Y., Bengio Y. (1995). Convolutional networks for images, speech, and time series. Handb. Brain Theory Neural. Netw..

[41] Tang J., Wang D., Zhang Z., He L., Xin J., Xu Y. (2017). Weed identification based on K-means feature learning combined with convolutional neural network. Comput. Electron. Agric..

[42] Sa I., Chen Z., Popović M., Khanna R., Liebisch F., Nieto J., Siegwart R. (2017). Weednet: Dense semantic weed classification using multispectral images and mav for smart farming. IEEE Robot. Autom. Lett..

[43] Milioto A., Lottes P., Stachniss C. Real-Time Semantic Segmentation of Crop and Weed for Precision Agriculture Robots Leveraging Background Knowledge in CNNs. Proceedings of the 2018 IEEE International Conference on Robotics and Automation (ICRA).

[44] Okamoto H., Murata T., Kataoka T., HATA S.I. (2007). Plant classification for weed detection using hyperspectral imaging with wavelet analysis. Weed Biol. Manag..

[45] Yang C., Prasher S.O., Landry J., DiTommaso A. (2000). Application of artificial neural networks in image recognition and classification of crop and weeds. Can. Agric. Eng..

[46] Ahmed F., Al-Mamun H.A., Bari A.H., Hossain E., Kwan P. (2012). Classification of crops and weeds from digital images: A support vector machine approach. Crop Prot..

[47] Haug S., Michaels A., Biber P., Ostermann J. Plant Classification System for Crop/Weed Discrimination without Segmentation. Proceedings of the IEEE Winter Conference on Applications of Computer Vision.

[48] Schmidhuber J. (2015). Deep learning in neural networks: An overview. Neural Netw..

[49] Krizhevsky A., Sutskever I., Hinton G.E. Imagenet Classification with Deep Convolutional Neural Networks. Proceedings of the Advances in Neural Information Processing Systems.

[50] Simonyan K., Zisserman A. (2014). Very deep convolutional networks for large-scale image recognition. arXiv.

[51] Szegedy C., Liu W., Jia Y., Sermanet P., Reed S., Anguelov D., Erhan D., Vanhoucke V., Rabinovich A. Going Deeper with Convolutions. Proceedings of the IEEE Conference on Computer Vision and Pattern Recognition.

[52] Szegedy C., Vanhoucke V., Ioffe S., Shlens J., Wojna Z. Rethinking the Inception Architecture for Computer Vision. Proceedings of the IEEE Conference on Computer Vision and Pattern Recognition.

[53] He K., Zhang X., Ren S., Sun J. Deep Residual Learning for Image Recognition. Proceedings of the IEEE Conference on Computer Vision and Pattern Recognition.

[54] Russakovsky O., Deng J., Su H., Krause J., Satheesh S., Ma S., Huang Z., Karpathy A., Khosla A., Bernstein M. (2015). Imagenet large scale visual recognition challenge. Int. J. Comput. Vis..

[55] Lee S.H., Chan C.S., Mayo S.J., Remagnino P. (2017). How deep learning extracts and learns leaf features for plant classification. Pattern Recognit..

[56] Yalcin H., Razavi S. Plant Classification Using Convolutional Neural Networks. Proceedings of the 2016 Fifth International Conference on Agro-Geoinformatics (Agro-Geoinformatics).

[57] Hedjazi M.A., Kourbane I., Genc Y. On Identifying Leaves: A comparison of CNN with Classical ML Methods. Proceedings of the 2017 25th Signal Processing and Communications Applications Conference (SIU).

[58] Ojala T., Pietikäinen M., Harwood D. (1996). A comparative study of texture measures with classification based on featured distributions. Pattern Recognit..

[59] Pietikäinen M., Hadid A., Zhao G., Ahonen T. (2011). Computer Vision Using Local Binary Patterns.

[60] Shan C., Gong S., McOwan P.W. (2009). Facial expression recognition based on local binary patterns: A comprehensive study. Image Vis. Comput..

[61] Brahnam S., Jain L.C., Nanni L., Lumini A. (2014). Local Binary Patterns: New Variants and Applications.

[62] Zhu C., Bichot C.-E., Chen L. (2013). Image region description using orthogonal combination of local binary patterns enhanced with color information. Pattern Recognit..

[63] Huang D., Shan C., Ardabilian M., Wang Y., Chen L. (2011). Local binary patterns and its application to facial image analysis: A survey. IEEE Trans. Syst. Man Cybern. Part C.

[64] Tan C., Sun F., Kong T., Zhang W., Yang C., Liu C. A Survey on Deep Transfer Learning. Proceedings of the International Conference on Artificial Neural Networks.

